# Characterization of PIVKA‐II Molecular Forms via Size Exclusion Chromatography and Hydrophobic Interaction Chromatography in HCC Patients

**DOI:** 10.1002/jcla.70114

**Published:** 2025-10-10

**Authors:** Yoshiyuki Kitamura, Katsumi Aoyagi

**Affiliations:** ^1^ Research and Development Division Fujirebio Inc. Tokyo Japan

**Keywords:** blood, hepatocellular carcinoma, hydrophobic interaction chromatography, immuno‐reactivity, PIVKA‐II

## Abstract

**Background:**

Protein induced by the absence of vitamin K or antagonist‐II (PIVKA‐II) is an important serological biomarker for the diagnosis of hepatocellular carcinoma (HCC). The crucial epitope of PIVKA‐II comprises a series of Glu residues located in the Glu‐Gla domain. The other antibody required for the PIVKA‐II sandwich immunoassay recognizes prothrombin fragments 1 and 2. However, the molecular diversity of these regions has not been well documented.

**Methods:**

PIVKA‐II immuno‐reactivities in blood taken from HCC patients were analyzed by size exclusion chromatography (SEC) and hydrophobic interaction chromatography (HIC). Obtained fractions were subsequently tested using MU‐3 monoclonal antibody (mAb) and a polyclonal anti‐prothrombin antibody (pAb). Immuno‐reactivity in each fraction was analyzed by an inhibition assay using prothrombin fragments and by a sandwich immunoassay.

**Results:**

SEC analysis of three paired serum and plasma samples gave a single peak of PIVKA‐II immuno‐reactivity corresponding to the expected molecular mass. However, HIC analysis using three paired sets and 14 HCC specimens revealed immuno‐reactivity present in two distinct peaks of either low (peak‐L: an elution position of 300–500 mM ammonium sulfate) or high (peak‐H: an elution position of 100–300 mM ammonium sulfate) hydrophobicity or both peaks together. Immuno‐reactivity of peak‐L was inhibited by human prothrombin fragment‐1 and detected by mAbs that recognize prothrombin‐1. Immuno‐reactivity of peak‐H was inhibited by human prothrombin fragment‐2 and detected by mAbs for prothrombin‐2.

**Conclusions:**

These results demonstrate PIVKA‐II exhibits molecular diversity, suggesting its potential as a diagnostic tool. This finding will contribute to further understanding of HCC and improve diagnostic accuracy.

## Graphical abstract

PIVKA‐II comprises two heterogeneous molecular forms with different levels regarding hydrophobicity, and PIVKA‐II in serum and plasma exists as distinct molecular forms with varying levels.

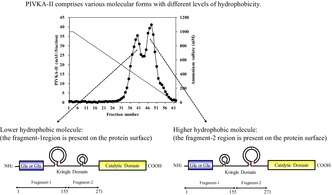

## 1 Introduction

Hepatocellular carcinoma (HCC) accounts for approximately 90% of primary liver cancers worldwide [1, 2]. The standard screening methods for the early diagnosis of HCC in high‐risk populations include ultrasonography, computed tomography (CT), magnetic resonance imaging (MRI), and serum tumor markers (TMs) [3]. However, although CT and MRI can significantly improve diagnostic accuracy of HCC, they are costly and therefore unsuitable for mass screening and surveillance [4]. Consequently, there is growing interest in the use of serum TMs for the early detection of hepatocellular carcinoma. The Japan Society of Hepatology recommends measurement of two or more TMs to diagnose small HCC [5]. Protein induced by vitamin K absence or antagonist II (PIVKA‐II) is an abnormal form of the coagulation protein prothrombin. It is also known as des‐gamma‐carboxy prothrombin (DCP) [6]. The level of PIVKA‐II is elevated in patients with HCC. However, PIVKA‐II levels are not correlated with alpha‐fetoprotein (AFP) levels or AFP‐L3 levels, which are other representative HCC biomarkers. Analysis of a combination of three tumor markers, AFP, AFP‐L3 fraction, and PIVKA‐II, has been proposed to enhance their diagnostic performance [7]. Therefore, PIVKA‐II can be used alongside AFP or AFP‐L3 as a complementary biomarker for HCC. Combining the two markers enhances HCC diagnosis sensitivity while minimizing specificity loss [6, 8, 9, 10]. Systematic reviews have shown that PIVKA‐II outperforms AFP in detecting HCC across diverse racial groups and sample sizes, as well as in cases of HBV‐related, HCV‐related, or mixed‐etiology HCC [11, 12]. Many studies have shown that serum PIVKA‐II levels are related to tumor size, vascular invasion, intrahepatic metastasis, and recurrence frequency after treatment. Therefore, PIVKA‐II can also be used as a prognostic predictor in patients with HCC [13, 14, 15, 16, 17]. According to tumor characterization, PIVKA‐II may play a crucial role in determining the most effective therapy.

To measure PIVKA‐II in blood, Motohara et al. established an immunoassay using MU‐3 monoclonal antibody (mAb) that specifically recognizes the Glu‐Gla domain [18, 19]. Subsequently, there have been numerous reports on the variation of the Glu‐Gla domain, including epitope analysis of the MU‐3 mAb by Naraki et al. [20], reactivity of the MU‐3 mAb with the number of Gla residues by Uehara et al. [21], and the relationship between various liver diseases and the number of Gla residues [22]. Indeed, distinct molecular forms of PIVKA‐II are known to exist according to the number of Glu groups, which can theoretically lead to 1023 variants. The Glu‐Gla domain of PIVKA‐II has been analyzed in detail by focusing on the different decarboxylation sites. Moreover, the clinical utility of PIVKA‐II has been assessed using MU‐3 mAb.

Recently, it was reported that PIVKA‐II is expressed in pancreatic adenocarcinoma cells. The molecular mechanisms involved in producing and secreting PIVKA‐II have been elucidated, suggesting that it has the potential to be a new biomarker [23, 24]. Although PIVKA‐II's emerging role as a biomarker beyond hepatocellular carcinoma (HCC) suggests broader clinical potential, detailed molecular characterization is essential to unlocking its full diagnostic utility. However, research focusing on molecular characteristics is limited. One such study, conducted by Kinukawa et al., reported that the 3C10 mAb recognizes the same epitope as the MU‐3 mAb and exhibits comparable immunoreactivity [25].

**FIGURE 1:**
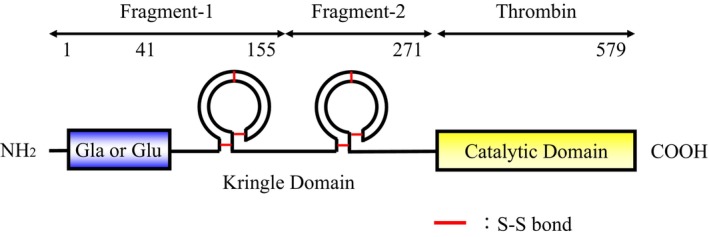
Domain structure of the PIVKA‐II molecule. PIVKA‐II is a protein with a molecular mass of 72 kDa that can be divided into three regions: Fragments 1, 2, and thrombin. Fragment‐1 contains a Glu‐Gla domain composed of 41 amino acids in the N‐terminal region.

As described above, the characteristics of the PIVKA‐II molecule have been analyzed mainly with a focus on the Glu‐Gla domain. The domain structure of the PIVKA‐II molecule is illustrated in Figure 1. However, the molecular diversity of PIVKA‐II in the prothrombin fragments 1 and 2 in the middle region, which are crucial epitopes for an immunoassay of PIVKA‐II, has not been studied in detail and remains largely unexplored. Addressing this gap could provide enhanced diagnostic tools and improve therapeutic decisions for HCC patients. Here, the aim of this study was to characterize PIVKA‐II molecular forms in HCC specimens.

## 2 Materials and Methods

### 2.1 Human Specimens and Reagents

Matched pairs of serum and EDTA‐2Na plasma samples from three patients were obtained from ProMedEX, LCC (Marlboro, NJ, USA), and 14 HCC serum samples were obtained from Trina Bioreactives AG (Zurich, Switzerland). Chemiluminescence enzyme immunoassays were performed using Lumipulse G PIVKA‐II reagent (Fujirebio Inc., Tokyo, Japan). The testing time of the fully automated LUMIPULSE G1200 analyzer was 30 min per assay. The Lumipulse G PIVKA‐II reagent comprised magnetic particles coated with anti‐PIVKA‐II mAb (MU‐3), which recognized an epitope within the Glu‐Gla domain at the N‐terminus of PIVKA‐II, and an alkaline phosphatase (ALP)‐conjugated anti‐prothrombin rabbit polyclonal antibody (pAb) made up in a buffer solution (50 mM Tris (hydroxymethyl) aminomethane‐HCl, 150 mM NaCl, 2% BSA, 0.05% TritonX‐100, 0.3 mM ZnCl_2_, 1 mM MgCl_2_, pH 6.8). The analyte, PIVKA‐II in the sample, is captured by MU‐3 mAb coated microparticles and then, after the washing step, the complex of analyte‐MU‐3 mAb coated microparticles is reacted with the ALP‐conjugated anti‐prothrombin pAb. A 20 μL sample was required for each assay. This method ensures high sensitivity and specificity in detecting PIVKA‐II levels.

### 2.2 Analysis of PIVKA‐II in HCC Samples by Size Exclusion Chromatography

Size exclusion chromatography (SEC) analysis of PIVKA‐II immuno‐reactivity was performed on a Superdex 200 10/300 column (Cytiva, Marlborough, MA, USA: 10 × 300 mm, particle size of 8.6 μm, fractionation range of 10–600 kDa). A 50 μL aliquot of sample was subjected to SEC. Each sample was eluted with 50 mM Tris (hydroxymethyl) aminomethane‐HCl buffer (pH 7.2) containing 150 mM NaCl, 0.1% NaN_3_. The flow rate was set to 0.5 mL/min at room temperature. After the separation, fractions were collected in 0.5 mL portions. The total amount of PIVKA‐II immuno‐reactivity in each fraction was then measured by Lumipulse G PIVKA‐II. The molecular mass of each fraction was estimated from a calibration curve obtained using standard proteins run under identical conditions (myoglobin (17 kDa), ovalbumin (44 kDa), gamma globulin (158 kDa), and thyroglobulin (670 kDa)).

### 2.3 Analysis of PIVKA‐II in HCC Samples by Hydrophobic Interaction Chromatography (HIC)

Serum and plasma samples from patients with HCC were conditioned using a 100 mM phosphate buffer pH 7.0 with 1 M (NH4)2SO4. A 50 μL aliquot of sample was injected onto a Phenyl Sepharose High Performance column (Cytiva: 6.2 × 33 mm, particle size of 34 μm). The washing buffer consisted of 100 mM phosphate buffer (pH 7.0) with 1 M (NH_4_)_2_SO4. The flow rate was maintained at 0.5 mL/min. The elution was performed using a gradient of ammonium sulfate concentration that decreased linearly to 0 mM (NH_4_)_2_SO_4_. The PIVKA‐II concentrations in each fraction were measured by Lumipulse G PIVKA‐II. This method aims to isolate specific components based on their hydrophobic interactions with the Phenyl Sepharose column. This HIC step is critical for analyzing the molecular diversity of PIVKA‐II and its diagnostic potential.

### 2.4 Inhibition Assay for Analysis of Immuno‐Reactivity in Each Fraction Obtained by HIC

The inhibition assay was performed using human prothrombin fragment‐1 antigen, human prothrombin fragment‐2 antigen, and human prothrombin fragment‐1/2 antigen (Prolytix, Essex Junction, VT, USA) containing two Kringle domains. In each assay, 30 μg/mL of antigen was added to the conjugate diluent to analyze the binding of ALP‐conjugated anti‐prothrombin pAb with various molecular forms of PIVKA‐II in the different elution fractions obtained through HIC.

### 2.5 Reactivity of Different mAbs to HIC Elution Fractions

Three anti‐prothrombin mAbs (hPTN7‐2, PT5‐23, and PT5‐42) were established by Fujirebio Inc., and two mAbs (BDI095 and AHP‐5013) were purchased from Santa Cruz Biotechnology (Dallas, TX, USA). These antibodies were conjugated with ALP based on the method detailed in [26] and diluted to 0.5 μg/mL with conjugate diluent. HIC elution fractions were analyzed to identify binding affinities and molecular diversity among anti‐prothrombin mAbs on a Lumipulse G1200 system using standard reagents combined with MU‐3 mAb coated microparticles and ALP conjugated to each anti‐prothrombin mAb. The chemiluminescent counts in each assay were then evaluated.

### 2.6 Epitope Analysis of Each mAb for Prothrombin

Assays were performed in 96‐well microtiter plates (Thermo Fisher Scientific, Waltham, MA, USA). Plates were first coated with Neutravidin (Pierce, Rockford, IL, USA) at a concentration of 5 μg/mL in coating buffer (0.1 M carbonate–bicarbonate buffer, pH 9.6) overnight at 4°C. The assay was conducted by incubating each well in 5 μg/mL of biotinylated synthetic peptide. The peptide comprised fragments of PIVKA‐II along with prothrombin fragment‐1 and prothrombin fragment‐2. This was done in phosphate‐buffered saline (PBS) pH 7.4 for 30 min at room temperature. After washing, blocking buffer (PBS containing 2% BSA, pH 7.4) was added for 60 min at room temperature before adding 50 μL of each mAb (5 μg/mL) per well. The reaction was allowed to proceed for 60 min at room temperature. After washing, 50 μL of ALP‐conjugated anti‐mouse IgG (Thermo Fisher Scientific; dilution ratio of 1:5000) was added to each well for 60 min at room temperature in assay buffer. The plates were then washed three times with wash buffer, and the enzyme color reaction was developed by adding 50 μL of substrate solution (10 mM *p*‐nitrophenyl phosphate (pNPP), 1 mM MgCl_2_, 1 M diethanolamine (DEA)‐HCl buffer, pH 10.5) for 30 min at room temperature. The reaction was then stopped by adding 50 μL of 0.1 M PBS pH 7.0 containing 50 mM EDTA. To quantify the binding efficiency, the absorbance of each well was measured at 450 nm (versus 630 nm as the reference wavelength) using an iMark Microplate Reader (BioRad).

## 3 Results

### 3.1 The Molecular Characteristics of PIVKA‐II in Serum and Plasma by SEC

**FIGURE 2:**
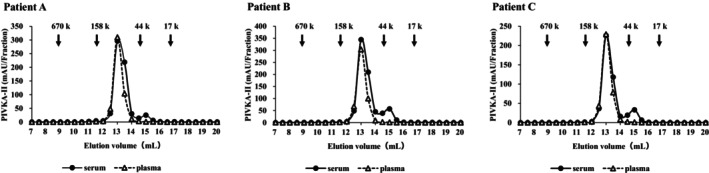
SEC analysis of HCC samples using a Superdex 200 10/300 column. Immunoreactive PIVKA‐II in each fraction was tested using Lumipulse PIVKA‐II. PIVKA‐II immunoreactivity in plasma samples was found to have an apparent molecular mass of 72 kDa. SEC analysis of serum samples gave two peaks: a major peak of 72 kDa and a minor peak of 40 kDa. The minor peak corresponded to a truncated form of PIVKA‐II after cleavage of the thrombin domain.

The molecular mass of PIVKA‐II in serum and plasma was estimated using SEC. Paired serum and EDTA‐2Na plasma samples from three HCC patients (Patients A–C) were analyzed. Serum PIVKA‐II values for patient A, B, and C were 50,403, 54,517, and 30,451 AU/mL, respectively. The main PIVKA‐II immuno‐reactivity was obtained at an elution volume corresponding to 72 kDa in both serum and plasma samples for all patients (Figure 2). The experimentally determined molecular mass corresponded to the anticipated mass of PIVKA‐II. In addition, a minor secondary immuno‐reactivity was identified in serum samples, arising from a degraded PIVKA‐II product with an apparent molecular mass of 30–40 kDa. This molecular species was considered to be a fragment of PIVKA‐II generated after the thrombin region had been cleaved. Indeed, a previous study has suggested that human prothrombin undergoes enzymatic digestion by serine proteases activated in the coagulation cascade [27].

### 3.2 The Molecular Characteristics of PIVKA‐II in Serum and Plasma by HIC

**FIGURE 3:**
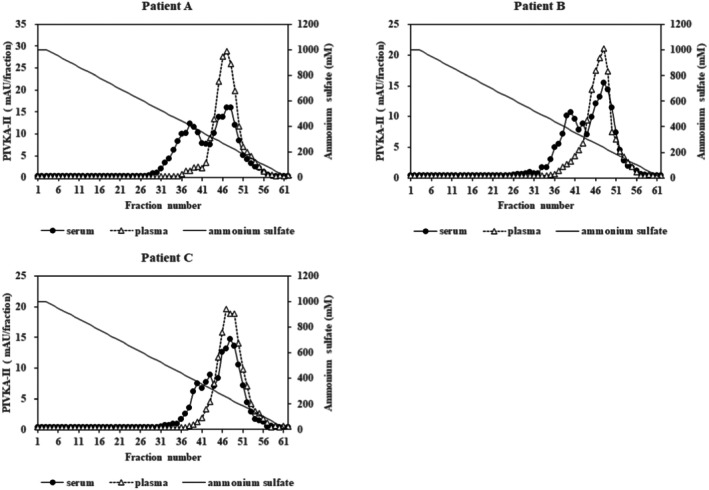
Analysis of PIVKAII in serum and plasma samples from patients with HCC using a HiTrap Phenyl HP column. Each serum and plasma sample from three patients was fractionated on the HIC. Fractions were analyzed for PIVKA II immunoreactivity using a combination of MU‐3 mAb and pAb‐ALP conjugate. The solid line with filled circles indicates PIVKA‐II immunoreactivity in serum samples, and the dotted line with open triangles indicates its immunoreactivity in plasma samples. Ammonium sulfate concentration is depicted by the plain solid line. The PIVKAII immunoreactivity in the plasma samples of these three patients mainly eluted at ammonium sulfate concentrations of 100–300 mM, whereas the PIVKAII immunoreactivity in the serum samples eluted as two main peaks at ammonium sulfate concentrations of 300–500 mM and 100–300 mM.

Next, the paired serum and EDTA‐2Na plasma samples used in SEC analysis were fractionated by HIC (Figure 3). In all three plasma samples, the PIVKA‐II immuno‐reactivities were detected at an elution position of 100–300 mM ammonium sulfate (peak‐H: higher hydrophobic fraction). Peak‐H profiles were very similar for each sample. However, in serum samples, a second PIVKA‐II immuno‐reactivity was also observed at an elution position of 300–500 mM ammonium sulfate (peak‐L: lower hydrophobic fraction) in addition to peak‐H. Notably, the fractionation patterns of peak‐L were different among each serum sample (Figure 3). These results indicate that PIVKA‐II in serum and plasma exists as distinct molecular forms with varying levels of hydrophobicity, but no significant difference in molecular mass.

**FIGURE 4:**
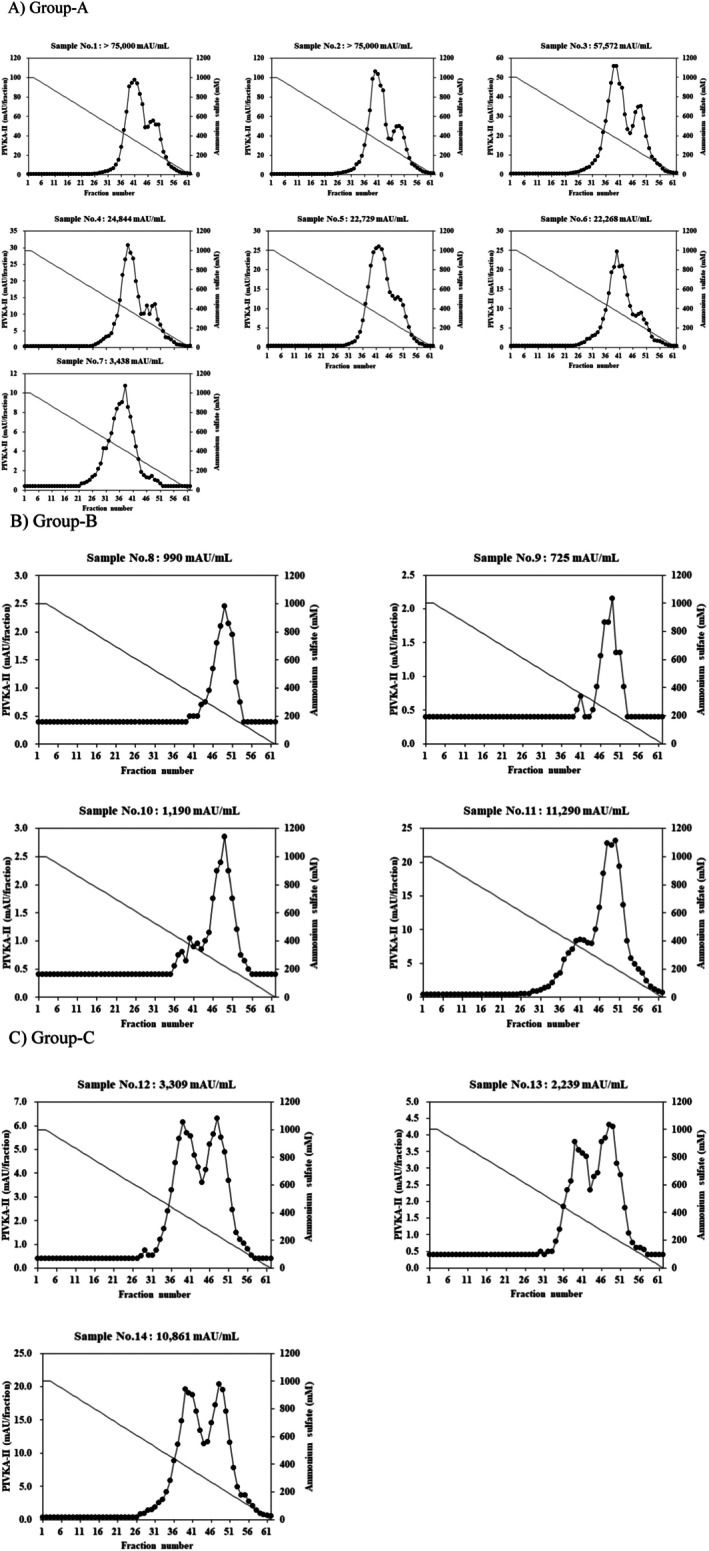
HIC analysis of 14 HCC samples using a HiTrap Phenyl HP column. Analysis revealed the samples could be classified into three groups (A–C) based on the shape of the profiles. Group A corresponded to the hydrophilicity‐rich fraction (A); Group B corresponded to the hydrophobicity‐rich fraction (B); Group C comprised both hydrophilicity‐ and hydrophobicity‐rich fractions (C). The solid line and closed circle profiles indicate immunoreactive PIVKA‐II. The solid line indicates ammonium sulfate concentration. Immunoreactive PIVKA‐II in each fraction was tested using Lumipulse G PIVKA‐II.

To confirm the hydrophobic variation of PIVKA‐II immuno‐reactivity in serum samples, an additional 14 serum samples taken from HCC patients were analyzed by HIC. To ensure 80% statistical power, a sample size calculation was performed in this study, determining that a minimum of twelve samples was necessary. This analysis revealed three different elution patterns among the samples (Figure 4). Each sample was classified into one of the three groups based on the characteristics of the elution profile. For the group A samples (sample No. 1–8), PIVKA‐II immuno‐reactivities were found in the fractions corresponding to the lower hydrophobic fraction (peak‐L) (Figure 4A). In contrast, for group B samples (sample No. 9–11), the immunoreactivities were observed in the fractions corresponding to higher hydrophobic fractions (peak‐H) (Figure 4B). In addition, group C samples (sample No. 12–14) showed PIVKA‐II immuno‐reactivities in fractions corresponding to both the peak‐L and the peak‐H (Figure 4C). These results indicated that PIVKA‐II in serum exists as various molecular forms that display different degrees of hydrophobicity among samples. PIVKA‐II values of the samples used in this analysis are shown on each figure.

The PIVKA‐II levels of peak‐H and peak‐L eluted by HIC and the corresponding ammonium sulfate concentration are given in Table 1. Peak‐H was distributed in the range of 339–407 mM ammonium sulfate, and peak‐L was distributed in the range of 203–237 mM. As a result of statistical analysis, the variations of each eluted peak were CV 5.2% (average: 376 mM) and CV 4.5% (average: 224 mM), respectively. This reproducibility highlights the reliability of HIC for analyzing molecular hydrophobicity differences in PIVKA‐II. This excellent reproducibility meant that its peak‐H and peak‐L were eluted at a consistent position regardless of the samples. These findings confirm that serum PIVKA‐II exhibits molecular diversity based on hydrophobic properties, emphasizing its complex nature in HCC patients.

**TABLE 1:** PIVKA‐II values of HCC samples used for HIC analysis were shown.

Group	Sample No.	PIVKA‐II values (mAU/mL)	PIVKA‐II levels (mAU/fraction)		Ammonium sulfate concentration (mM)	
			peak‐L: lower hydrophobic fraction	peak‐H: higher hydrophobic fraction	peak‐L: lower hydrophobic fraction	peak‐H: higher hydrophobic fraction
A	No. 1	> 75,000	98	56	356	237
	No. 2	> 75,000	106	50	373	220
	No. 3	57,572	56	35	390	220
	No. 4	24,844	31	13	390	220
	No. 5	22,729	26	13	339	220
	No. 6	22,268	25	9	373	220
	No. 7	3438	11	N.A	407	N.A
B	No. 8	990	N.A	2	N.A	220
	No. 9	725	1	2	356	220
	No. 10	1190	1	3	373	220
	No. 11	11,290	9	23	356	203
C	No. 12	3309	6	6	390	237
	No. 13	2239	4	4	390	237
	No. 14	10,861	20	20	390	237

*Note:* In addition, the PIVKA‐II levels of peak‐L or peak‐H eluted by HIC and the ammonium sulfate concentration corresponding to their peaks were shown.

### 3.3 Inhibition Assay Using Human Prothrombin Fragment‐1 and ‐2 Antigen

**FIGURE 5:**
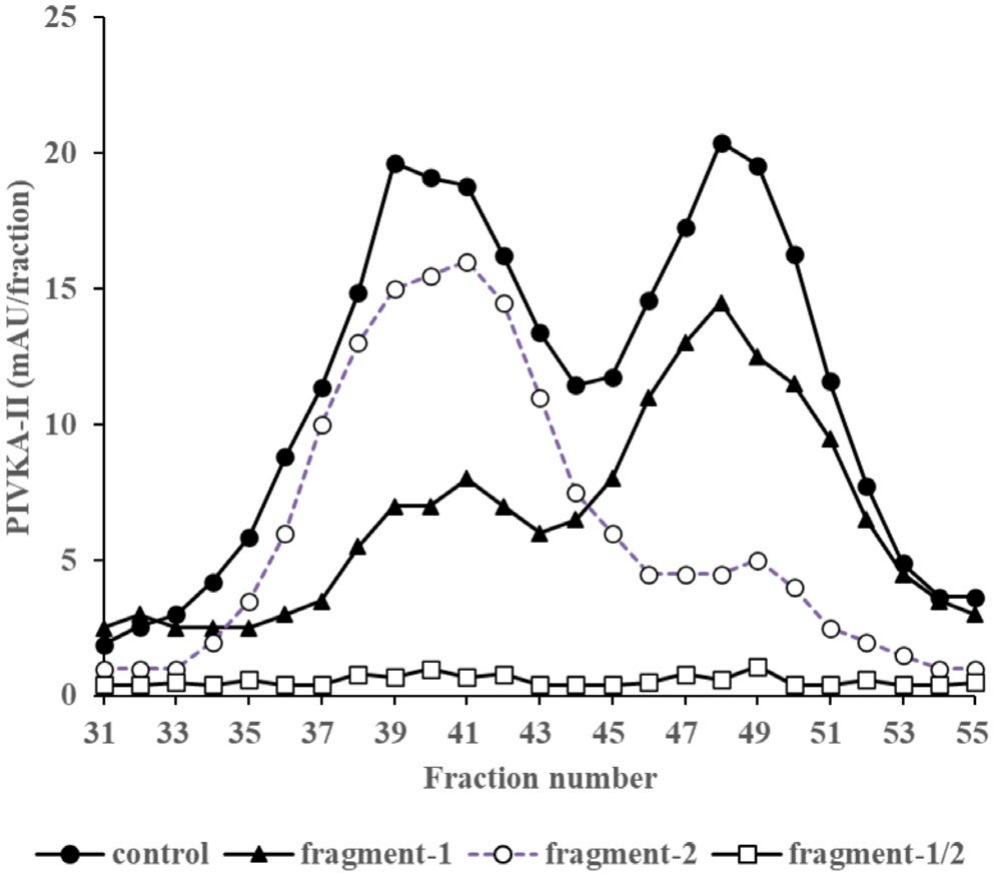
Inhibition assay using human prothrombin fragment‐1 and human prothrombin fragment‐2 antigen. Elution profiles of Sample No. 14 from group C. The solid line with closed circle profile indicates immunoreactive PIVKA‐II without inhibition by the antigen (control). The solid line with closed triangle profile indicates immunoreactive PIVKA‐II in the inhibition assay with human prothrombin fragment‐1. The dashed line with open circle profile indicates immunoreactive PIVKA‐II in the inhibition assay with human prothrombin fragment‐2. The solid line with open square profile indicates immunoreactive PIVKA‐II in the inhibition assay with human prothrombin fragment‐1/2.

To better understand the molecular structure and diagnostic potential of PIVKA‐II, its immuno‐reactivity patterns were analyzed in relation to specific molecular domains. Specifically, each HIC fraction of sample No. 14, which included both peak‐L and peak‐H, was examined to determine the relationship between immuno‐reactivity and the domains in the PIVKA‐II molecule. Inhibition assays were performed in which human prothrombin fragment‐1, ‐2, or ‐1/2 were included in the conjugate diluent (Figure 5). In almost all fractions, the immuno‐reactivity for PIVKA‐II was inhibited by prothrombin fragment‐1/2. Notably, the lower hydrophobic fractions (peak‐L) showed inhibition with fragment‐1, while the higher hydrophobic fractions (peak‐H) were mainly inhibited by fragment‐2. These results indicated that the PIVKA‐II immuno‐reactivity of peak‐L and peak‐H reflected the fragment‐1 and fragment‐2 regions, respectively.

### 3.4 Reactivity of mAbs for Prothrombin in the HIC Elution Fractions

To further evaluate the molecular characteristics of PIVKA‐II, we examined its immuno‐reactivity in the HIC elution fractions of sample No. 14 using various mAbs. Initially, we performed epitope analysis of the mAbs using prothrombin fragment‐1, fragment‐2, and each biotinylated synthetic peptide as described in materials and methods. hPTN7‐2, PT5‐23, and PT5‐42 reacted with fragment‐1, and BDI095 and AHP‐5013 reacted with fragment‐2. In the analysis using synthetic peptides, only AHP‐5013 recognized the two peptides corresponding to amino acids 145–164 and amino acids 157–176, as well as prothrombin fragment‐2 (Table 2). MU‐3 mAb displayed reactivity to the PIVKA‐II synthetic peptide corresponding to amino acids 13–32. However, it showed no reactivity to prothrombin fragment‐1. Note that mAbs with the epitopes to both fragment‐1 and fragment‐2 were included among the selected 5 mAbs. This allowed for a comparative analysis of reactivity to the fragment‐1 and fragment‐2 using these mAbs.

**TABLE 2:** Epitope analysis of each mAb using peptides comprising 20 amino acids of PIVKA‐II.

	Amino acid number	MU‐3	pAb	hPTN7‐2	PT5‐23	PT5‐42	BDI095	AHP‐5013
PIVKA‐II synthetic peptide	1–20	−	−	−	−	−	−	−
	13–32	+ +	−	−	−	−	−	−
	25–44	−	−	−	−	−	−	−
	37–56	−	−	−	−	−	−	−
	49–68	−	+	−	−	−	−	−
	61–80	−	−	−	−	−	−	−
	73–92	−	−	−	−	−	−	−
	85–104	−	−	−	−	−	−	−
	97–116	−	+	−	−	−	−	−
	109–128	−	−	−	−	−	−	−
	121–140	−	−	−	−	−	−	−
	133–152	−	−	−	−	−	−	−
	145–164	−	+	−	−	−	−	+ +
	157–176	−	+	−	−	−	−	+ +
	169–188	−	−	−	−	−	−	−
	181–200	−	−	−	−	−	−	−
	193–212	−	−	−	−	−	−	−
	205–224	−	+	−	−	−	−	−
	217–236	−	−	−	−	−	−	−
	229–248	−	+	−	−	−	−	−
	241–260	−	−	−	−	−	−	−
	253–271	−	+	−	−	−	−	−
Prothrombin fragment‐1	1–155	−	+ +	+ +	+ +	+ +	−	−
Prothrombin fragment‐2	156–271	−	+ +	−	−	−	+	+

*Note:* A library of 22 overlapping synthetic peptides (20 amino acids) with an offset of 8 residues covering the region 1 to 271 (fragment‐1 and 2) of PIVKA‐II was used. In addition, two human prothrombin fragments were also included. Reactivities of mAb MU‐3, hPTN7‐2, PT5‐23, PT5‐42, BDI095, AHP‐5013, and pAb were verified.

**FIGURE 6:**
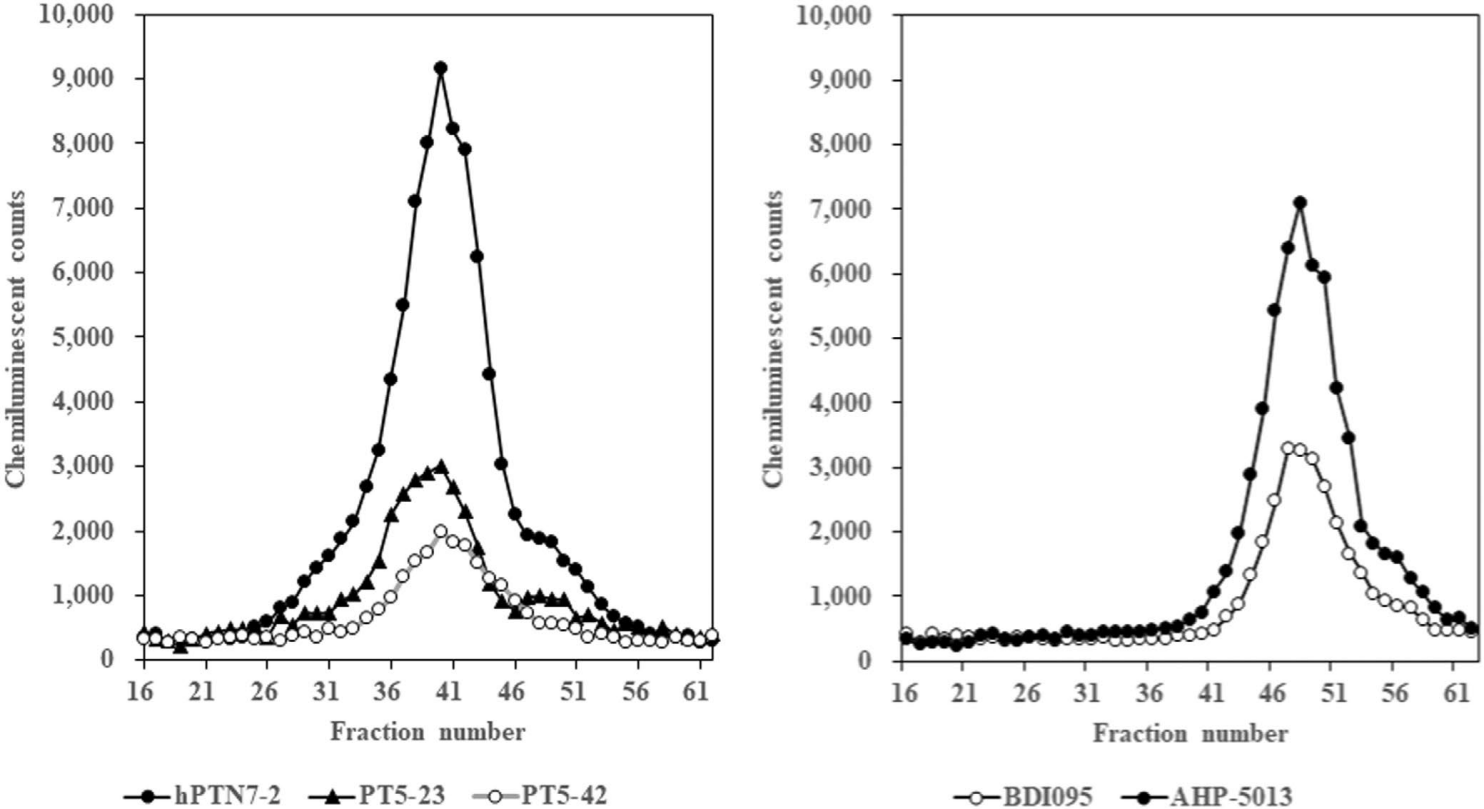
Evaluation of mAb reactivity in the elution profiles of Sample No. 14 (Group C). Each colored profile indicates immunoreactive PIVKA‐II. Immunoreactive PIVKA‐II in each fraction was tested using reagents combined with anti‐MU‐3 mAb‐coated magnetic particles and ALP‐labeled anti‐prothrombin mAbs.

In sandwich immunoassays using these mAbs instead of pAb, immuno‐reactivity in the HIC elution fractions of sample No. 14 (used in Figure 5) was examined. Immunoassays using three antibodies (hPTN7‐2, PT5‐23, and PT5‐42), which recognized fragment‐1, showed immuno‐reactivity to the lower hydrophobic fractions (peak‐L). By contrast, immunoassays using two antibodies (BDI095 and AHP‐5013), which recognized fragment‐2, showed immuno‐reactivity to the higher hydrophobic fractions (peak‐H) (Figure 6). These results confirmed that peak‐L and peak‐H fractions obtained by HIC corresponded to PIVKA‐II immuno‐reactivity to the fragment‐1 and ‐2 region, respectively. In addition, although pAb for PIVKA‐II could detect the immuno‐reactivity of fragment‐1 and ‐2, the results using mAb indicated that a combination of mAbs that recognized both fragment‐1 and ‐2 was required.

## 4 Discussion

Currently, PIVKA‐II is routinely measured by various immunoassays in the clinical laboratory, including the chemiluminescent enzyme immunoassay (CLEIA). Most reagents for PIVKA‐II utilize MU‐3 mAb as the solid phase antibody, with either mAbs or pAbs against prothrombin as the antibodies for detection [28, 29, 30]. In this study, we analyzed PIVKA‐II immuno‐reactivity in serum and plasma using SEC and HIC, which was detected by immunoassay using MU‐3 mAb and anti‐prothrombin pAb.

Analysis of both sample types by SEC showed PIVKA‐II immuno‐reactivity as a main peak corresponding to an apparent molecular mass of about 75 kDa. This value agreed with the theoretical mass of PIVKA‐II including fragments‐1, ‐2, and thrombin. In addition, SEC analysis of serum samples also gave a small peak corresponding to a lower molecular mass of 30–40 kDa, which is presumed to be the truncated fragment of PIVKA‐II after the thrombin region had been cleaved [27]. However, this truncated form of PIVKA‐II comprised only a minor component of the sample.

Next, the results of HIC analysis revealed that PIVKA‐II immuno‐reactivity in serum and plasma had different hydrophobic characteristics. PIVKA‐II in plasma was found to be very hydrophobic, and its molecular properties were highly consistent among plasma samples (Figure 3). By comparison, PIVKA‐II in serum displayed striking levels of molecular diversity. Specifically, serum PIVKA‐II showed either lower hydrophobicity, higher hydrophobicity, or both types (Figures 3 and 4).

Considering the results of the analysis by SEC and HIC, the marked difference in hydrophobicity of PIVKA‐II could not be due to the modification of any functional group or protein interaction, or at least not those that would significantly alter the molecular mass. Rather, the variation in hydrophobicity must be due to the diverse characteristics of the PIVKA‐II molecules alone.

Further analysis showed PIVKA‐II immuno‐reactivity in eluted fractions of lower hydrophobicity that were inhibited by the human prothrombin fragment‐1 antigen (Figure 5). Conversely, eluted fractions of higher hydrophobicity were inhibited by the human prothrombin fragment‐2 antigen (Figure 5). These results suggested that fractions of lower hydrophobicity corresponded to reactivity with the fragment‐1 region, whereas fractions of higher hydrophobicity corresponded to reactivity with the fragment‐2 region.

The study using mAbs against prothrombin revealed that three antibodies, hPTN7‐2, PT5‐23, and PT5‐42, were reactive to the lower hydrophobic fractions (Figure 6). Two antibodies, BDI095 and AHP‐5013, were reactive to higher hydrophobic fractions (Figure 6). Our analysis confirmed that hPTN7‐2, PT5‐23, and PT5‐42 recognize epitopes located in the fragment‐1 region, whereas BDI095 and AHP‐5013 recognize epitopes on the fragment‐2 region. As a result of epitope mapping, all antibodies were found to react with recombinant antigens present in the fragment‐1 or ‐2 region, but only AHP‐5013 was able to recognize the linear sequence. These observations indicated that the antibodies recognize conformational epitopes. Taken together, these results suggest that the region containing the Kringle domain of PIVKA‐II is also likely to undergo conformational changes, which may lead to different molecular characteristics. Indeed, PIVKA‐II could exist in a conformation in which either the fragment‐1 or ‐2 region is present on the protein surface, or both regions are present on the protein surface. Thus, the conformational changes could account for the variation of molecular forms with distinct levels of hydrophobicity. The detection of PIVKA‐II of various molecular forms could be established by using mAbs that react with fragment‐1 and ‐2 and by optimizing the reactivity to each fragment. In summary, our study has demonstrated that PIVKA‐II exists in a diverse range of molecular forms related to the surface accessibility of the fragment‐1 and ‐2 regions in addition to the well‐documented variations within the Glu‐Gla region.

We can measure PIVKA‐II in plasma by using an antibody that recognizes fragment‐2. However, measuring PIVKA‐II in serum requires antibodies that recognize both fragment‐1 and ‐2. Thus, using an inappropriate antibody for detecting PIVKA‐II in serum may generate spurious results. For example, one approach could be to use pAb, which widely cover epitopes on fragment‐1 and ‐2, for measuring PIVKA‐II. The polyclonal antibodies generally have the advantages of high affinity for the antigen and high ability to capture the target protein because they can recognize multiple epitopes, but they also have disadvantages such as difficulty in mass producing the same antibody, insufficient reproducibility between batches, and large lot‐to‐lot differences compared to monoclonal antibodies. However, as mentioned above, serum PIVKA‐II molecules are highly diverse, and it is difficult to achieve a consistent balance of antibodies reacting to the fragment‐1 and ‐2 regions among pAb lots. For that, using mAbs would be another way to make a breakthrough. If only mAbs that recognize either fragment‐1 or ‐2 are used, it may not be possible to develop an accurate quantitative assay for PIVKA‐II, and discrepancies may occur, especially in serum and plasma PIVKA‐II assays. Therefore, from the point of view of the accurate assay, it is reasonable to use two types of mAbs.

This study has demonstrated that the PIVKA‐II molecule exists in multiple forms, as indicated by our HIC analysis. Intriguingly, samples with relatively high levels of PIVKA‐II tended to have an eluted peak of lower hydrophobicity (Figure 4 and Table 1). However, the apparent relationship between the level of PIVKA‐II and its hydrophobic nature remains unclear. Since HCC arises from various factors such as infection with HBV or HCV, alcoholic liver disease, and non‐alcoholic steatohepatitis, it is quite interesting to analyze whether the different etiologies are relevant for the molecular character. A significant difference in cut‐off values for HCC diagnosis and the distribution of PIVKA‐II levels across ethnicities and sexes has been well documented [31, 32, 33]. Recently, it has been reported that PIVKA‐II serves as a valuable prognostic predictor, transplantation eligibility, resectability, tumor recurrence, therapeutic efficacy, and malignant tumor behaviors [34] and a dual‐positive biomarker model of AFP‐L3 ≥ 15% and DCP ≥ 7.5 ng/mL strongly predicts the risk of early HCC recurrence [35]. Although this study was only focused on the diverse characteristics of PIVKA‐II molecules in HCC patients, the comprehensive comparative study, including race, sex, etc., might lead to another aspect of molecular characteristics. Furthermore, considering the future clinical application, it would be necessary to analyze the relationship in terms of tumor size, stage, and grade of HCC and the relevance to recurrence and prognosis. Monitoring the heterogeneity of PIVKA‐II molecules may provide valuable diagnostic and therapeutic insights for HCC patients. Therefore, it would be interesting to investigate PIVKA‐II molecular variations across different HCC etiologies, incorporating advanced proteomic techniques to comprehensively map molecular diversity and its clinical implications in a future study.

## 5 Conclusion

The diverse molecular forms of PIVKA‐II in blood samples present a significant step toward enhancing diagnostic precision and patient care in HCC. We have demonstrated that PIVKA‐II consists of various molecular forms in blood samples, influenced by variations in prothrombin fragments 1 and 2. Notably, the molecular characteristics in serum and plasma were found to differ. These findings pave the way for a more robust PIVKA‐II assay, contributing to accurate diagnosis and improved follow‐up care for HCC patients. This understanding sets the stage for future studies to explore how these molecular forms correlate with disease progression and treatment outcomes.

## Author Contributions

All authors have contributed to the writing and editing of the manuscript, have accepted responsibility for the entire content of this submitted manuscript, and have approved submission.

## Consent

The authors have nothing to report.

## Conflicts of Interest

Y. Kitamura is an employee of Fujirebio Inc. K. Aoyagi is a board member of Fujirebio Inc. Fujirebio Inc. holds patents (PCT/JP2016/079801) on the methods of [PIVKA‐II assay method and method for manufacturing reagent or kit FOR PIVKA‐II immunoassay]. Y. Kitamura and K. Aoyagi are the inventors of the patent.

## Data Availability

The data that supports the findings of this study is available on request from the corresponding author.
